# Traumatic Reticuloperitonitis in Water Buffalo (*Bubalus bubalis*): Clinical Findings and the Associated Inflammatory Response

**DOI:** 10.1155/2013/808656

**Published:** 2013-07-30

**Authors:** Maged El-Ashker, Mohamed Salama, Mohamed El-Boshy

**Affiliations:** ^1^Department of Internal Medicine and Infectious Diseases, Faculty of Veterinary Medicine, Mansoura University, Mansoura 35516, Egypt; ^2^Department of Biochemistry, Faculty of Veterinary Medicine, Mansoura University, Mansoura 35516, Egypt; ^3^Department of Laboratory Medicine, Faculty of Applied Medical Science, Umm Al-Qura University, Makkah 21955, Saudi Arabia

## Abstract

The present study was carried out to describe the clinical picture of traumatic reticuloperitonitis (TRP) in water buffalo (*Bubalus bubalis*) and to evaluate the inflammatory and immunologic responses for this clinical condition. Twenty-two buffalo with acute local TRP were monitored in our study. Additionally, 10 clinically healthy buffalo were randomly selected and served as controls. Acute local TRP was initially diagnosed by clinical examination and confirmed by ultrasonographic (USG) examination and/or necropsy findings. Blood samples were collected from all examined buffalo to measure the respective levels of tumor necrosis factor alpha (TNF-*α*), interleukin (IL)-1*β*, IL-6, IL-10 and interferon gamma (INF)-*γ*, serum amyloid A (SAA), C-reactive protein (CRP), haptoglobin (Hp), fibrinogen (Fb), and serum sialic acid (SSA). It was found that TNF-*α*, IL-1*β*, IL-6, IL-10, SAA, CRP, Hp, Fb, and SSA were significantly higher in buffalo with TRP than the controls. Our findings suggest that the examined immunologic variables were helpful in documenting the inflammatory response in buffalo with TRP. However, their diagnostic usefulness only becomes apparent when considered in tandem with the clinical findings for any given animal, its anamnesis, and a subsequent USG assessment. Due to the frequent complications of TRP, more accurate indicators of its occurrence and severity would be useful.

## 1. Introduction

Water buffalo (*Bubalus bubalis*) have a special importance in milk and meat production in the Nile River valley in Egypt [1] where the species can compete successfully and surpass the cattle in its ability to adapt to hot climates and swampy landscapes [2]. Insults and infections of the bovine forestomach from ingested foreign bodies are relevant worldwide and are economically significant due to livestock fatalities and production losses. Traumatic reticuloperitonitis (TRP) and allied syndromes are among the most commonly occurring diseases of the digestive tract of bovines that have been a concern for veterinarians over many years [3]. Bovines are more likely to ingest foreign bodies than small ruminants since they do not use their lips for prehension and will ingest foreign bodies in feedstuffs that may puncture or perforate the reticulum. The risk and sequelae of TRP syndrome are considerably higher in buffalo than in cattle [4] and extremely common within developing countries, possibly due to less organized small-scale farming and the unsatisfactory standards of animal management and feeding regimes.

The clinical signs associated with TRP are consistent with an acute localized peritonitis including anorexia, fever, tachypnea, and lordosis with abducted elbows [5, 6]. The diagnosis of TRP is challenging because the development of this syndrome is a complex process and could coincide with other syndromes [7]. However, the initial diagnosis of TRP is primarily based upon clinical signs and laboratory findings and confirmed by radiography and ultrasonography [5, 6].

Acute phase proteins (APPs) have become a focus for researchers in human diagnostic medicine and have recently been identified in various animal species [8]. The most commonly investigated APPs in bovines are haptoglobin (Hp), serum amyloid A (SAA), C-reactive protein (CRP), fibrinogen (Fb), ceruloplasmin, alpha 1-antitrypsin, and alpha 1-acid glycoprotein [7, 9–11]. APPs are plasma proteins of hepatic origin that are mainly synthesized during the acute phase response (APR). APR is a component of the innate immune response and is observed uniformly across animal species. APR is initiated when animals are subjected to various external or internal challenges, including trauma, inflammation, infection, environmental stress factors, tumours, and immunological disorders [12]. Tissue at the site(s) of inflammation or infection release cytokines, including interleukin (IL)-1, IL-6, and tumor necrosis factor (TNF)-*α*. Such proinflammatory cytokines induce local and systemic reactions, typically pyrexia, leukocytosis, hormonal variations, and muscle protein depletion and stimulate the hepatic release of APPs [13].

Whilst APPs are considered to be components of the nonspecific innate immune response involved in the restoration of homeostasis and bacteriostasis, their serum concentrations are correlated with the severity of disease and the degree of tissue damage present in the affected animal [14]. The exact nature of APPs and the timescale for modifications of the proteins vary between species according to the aetiology and the underlying inflammatory process. Bacterial infections induce strong APRs whilst viral infections typically produce weak or occult acute phase reactions [15].

Sialic acids (SA) are widely distributed within animal tissue, occupying the terminal position on macromolecules and cell membranes, and are integral to numerous physiological and pathological processes [16, 17]. They are normally present in human and animal serum, although serum concentrations change under pathological conditions [16, 17]. In the literatures, the cytokine response in buffalo with TRP has not been described. The present study was therefore undertaken to investigate the clinical signs of TRP in water buffalo and to evaluate the inflammatory and immunological responses associated with the consequent pathology.

## 2. Materials and Methods

### 2.1. Study Population

Twenty-two water buffalo cows (*Bubalus bubalis*) were used in this study, where 4 were in the final stages of gestation and 18 were within 6 weeks postpartum: ages ranged from five to seven years. Additionally 10 clinically healthy periparturient buffalo cows between three and five years of age served as a control group. The buffalo were examined at the Veterinary Teaching Hospital, in Mansoura University's Faculty of Veterinary Medicine. The diseased buffalo exhibited signs of inappetence, recurrent tympany, decreased fecal output, falling milk yield, and mild pyrexia or euthermia. Acute local TRP was initially diagnosed from the anamnesis and a physical examination and confirmed by ultrasonographic (USG) examination and/or necropsy findings. Cases of chronic local TRP or diffuse peritonitis were not diagnosed during this investigation. Selection criteria were applied to the examined animals, excluding individuals with concomitant ailments and thereby removing experimental confounds. Excluded individuals exhibited signs of mastitis (*n* = 2), arthritis (*n* = 1), laminitis (*n* = 1), enteritis (*n* = 2), and bronchopneumonia (*n* = 1). Once diagnosis of acute local TRP had been made, medical and surgical managements were subsequently offered to owners. Conservative medical management included the immobilization of animals, the ingestion of a magnet, and the administration of broad spectrum antibiotics (penicillin and streptomycin by intramuscular injection twice daily at a dose of 22000 IU/Kg BW and 25 mg/Kg BW, resp.), as well as a non-steroidal anti-inflammatory drug (flunixin meglumine injected intravenously once daily at a dose of 2.2 mg/kg BW). Rehydrating intravenous fluids were also administered to patients on an individual basis. Where improvement in clinical signs had not occurred within 3 days of treatment, a rumenotomy was indicated and performed following the standard procedures. Follow-up information was collated via clinical examination, through owner contact, and via referring veterinarians regarding the health status of animals post treatment.

### 2.2. Clinical Examinations

 Both diseased buffalo and control animals were subjected to a full clinical examination, where the cardinal signs (including rectal temperature, heart rate, respiratory rate, and auscultation of the respiratory tract) were recorded. Abdominal ballottement and rectal palpation were also performed. Tests for a reticulum foreign body were implemented, including deep palpation of the abdominal wall caudal to the xiphoid, pinching of the withers, and placing a rod beneath the abdomen to elicit a grunt. A metal detector was also applied over the ventral and ventrolateral regions of the chest and abdomen to detect a ferromagnetic foreign body.

### 2.3. Sampling and Measurements

Upon admission, blood was collected from all examined animals through jugular venepuncture and placed, respectively, into heparinized tubes, tubes without anticoagulant, and tubes with EDTA-yielding samples for plasma, serum, and whole blood. Intravenous fluids were not administered to patients prior to blood sampling. Plasma was separated from heparinized blood by centrifugation at 3000 g for 10 minutes and kept frozen at −20°C until required, together with the collected serum.

The collected blood plasma was used for estimation of selected APPs such as SAA, CRP, Hp, and Fb by using the K-Assay kit (Kamiya Biomedical Company, USA) and an automated microplate ELISA reader (Bio TEC, ELX800G, USA) according to the manufacturer's instructions. Serum samples were used for estimation of TNF-*α*, IL-6 (Kamiya Biomedical Company, USA), IL-1*β* (Thermo Scientific Pierce Protein Biology Products-Rockford, Illinois), IL-10 (Genorise Scientific Inc., Philadelphia, USA), and IF-*γ* (BioSource Europe S.A., Rue de l'Industrie, Belgium) according to the manufacturers' instructions; the plates were read at 450 nm, and a correction wave length of 550 nm was measured on a computerized automated microplate ELISA reader (Bio TEC, ELX800G, USA). Values were expressed in picograms per millilitre (pg/mL). Samples were run in duplicate for all of the examined cytokines and APPs.

SA was measured in the serum samples according to the method described by Shamberger [18] by using Ehrlich's reagent (made by adding 0.7 g of p-dimethylaminobenzaldehyde (Sigma Aldrich) to 150 mL of concentrated HC1 and 100 mL of distilled water). The absorbance of samples was measured at 525 nm by using Jenway 7305, UV-VIS spectrophotometer (Jenway Scientific Equipment, UK). Data were expressed as mmol/L. Conversely, the whole blood samples were used for routine haematological examinations including total and differential leukocyte counts.

### 2.4. USG Examination

USG examination was performed whilst the animals were in standing position using 3.5 and 5.0 MHz transducers (Chison Medical imaging Co., Ltd., Wuxi, China 214142) as described by Braun [19]. For examination of the reticulum, the transducer was applied to the ventral aspect of the thorax on both sides of the sternum as well as to the left and right lateral thorax up to the level of the cubitus. Examination of each lung area was performed with the transducer held parallel to the ribs, from the third to the eleventh intercostal space.

Echocardiographic examinations were conducted while the buffalo were standing. The third, fourth and fifth intercostal spaces in the cardiac region were examined ultrasonographically on the right and the left sides of the thorax. The forelimbs were moved cranially to facilitate improved contact between the probe and the intercostal space. Within the cardiac region, the heart, major blood vessels, and the mediastinal region were imaged. Additionally, the tricuspid, mitral, pulmonary, and aortic valves were also scanned.

### 2.5. Gross Postmortem Examination (PM)

Four buffalo were subjected to complete PM examination (with approval of the Faculty Ethics Committee), and the results were recorded.

### 2.6. Statistical Analysis

Data were statistically analyzed using the Graphpad Prism software package (Graphpad Prism version 5.0, Graph Pad software Inc., San Diego). Means and standard errors of means (SEM) were estimated for each variable, and at *P* < 0.05 results were considered statistically significant. Significant correlations between the clinical variables and the occurrence of TRP were examined via the Chi-square test. 

## 3. Results

The clinical and laboratory findings for diseased and control buffalo are summarized in Tables 1, 2, and 3. In Table 1, heart rate, respiratory rate, and rectal temperature were significantly higher in diseased buffalo compared to the controls (*P* < 0.006, *P* < 0.001, *P* < 0.049, resp.). Moreover there were significant correlations between the incidence of TRP and (1) observed inappetence (*P* < 0.01), (2) recurrent tympany (*P* < 0.0001), (3) lacrimation (*P* < 0.0001), and (4) decreased milk yield (*P* < 0.0001); other clinical findings showed no significant correlations with the incidence of TRP. Haematologically, buffalo with TRP were presented with neutrophilia (*P* < 0.0131), elevated band cells (*P* < 0.0001), monocytosis (*P* < 0.0001), and lymphocytopenia (*P* < 0.0003) compared to controls: leukopenia and eosinopenia were not indicated (Table 2). Biochemically, it was found that TNF-*α*, IL-1*β*, IL-6, IL-10, SAA, CRP, Hp, Fb, and SA levels were significantly higher (*P* < 0.0003 for IL-1*β* and *P* < 0.0001 for the other variables) in buffalo with TRP compared to controls: serum IF-*γ* showed no significant difference between diseased buffalo and controls (Table 3). Six buffalo with TRP responded to conservative medical treatment whilst 12 required a rumenotomy.

### 3.1. USG Findings

USG examination revealed that the amplitude of the reticulum contraction in the diseased buffalo was reduced. Furthermore, the frequency of the reticulum contractions was also decreased to one or zero contractions every two minutes, and, if present, the contractions were only 1–3 cm: where extensive adhesions were present, no reticulum contractions were observed. Deposits of fibrin interspersed with fluid pockets were frequently seen between the reticulum, the dorsal ruminal sac, and the diaphragm.

### 3.2. Necropsy Findings

**Figure 1:**
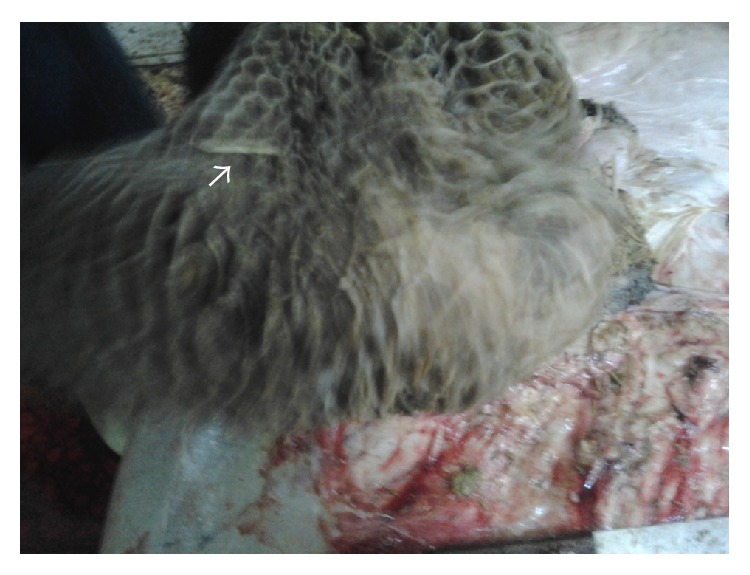
Buffalo with TRP showing foreign body perforating the reticulum.

**Figure 2:**
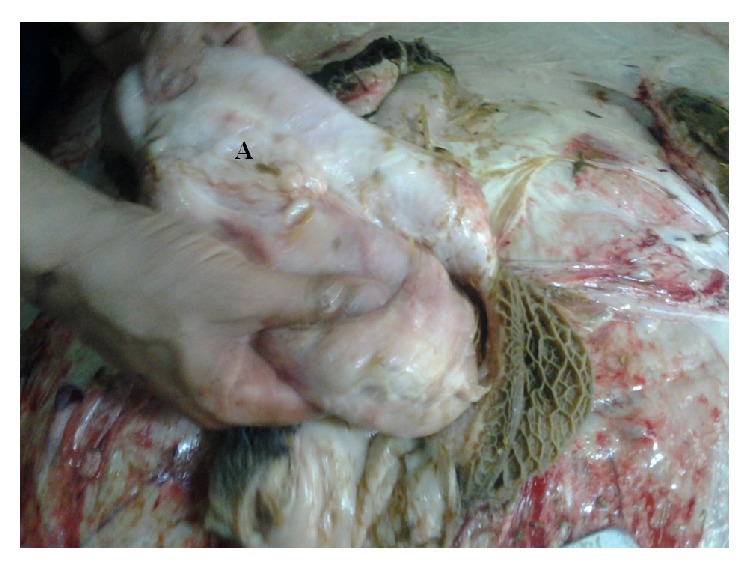
Buffalo with traumatic TRP showing peri-reticular abscess (A).

PM examination of deceased buffalo revealed that foreign bodies (including pieces of wire and nails) had perforated the reticular wall (Figure 1), forming peri-reticular abscess (Figure 2) and were embedded in the diaphragm.

## 4. Discussion

TRP has become increasingly common and is particularly challenging to manage as its clinical presentation is often vague. An initial diagnosis of TRP in diseased buffalo was achieved upon the basis of clinical signs and laboratory findings and confirmed by USG evaluation and/or necropsy. The clinical findings of TRP in examined buffalo were consistent with the literatures [5, 6]. Indications of algesia associated with early TRP in cattle [20] (including stiff gait, lordosis, and painful urination/defecation) were less common in diseased buffalo. Hence, early diagnosis of TRP becomes more difficult in buffalo compared to cattle, and these findings are consistent with the literatures [5, 6].

APPs play a major role in the innate immune response involving physical and molecular responses that serve to both prevent inflammation and initiate the inflammatory processes, as well as clear potential pathogens, and contribute to healing [8]. To date, the changes in acute phase cytokines in buffalo with TRP have not previously been described in the literatures. We hypothesized that TRP in buffalo can be associated with cytokine production. Indeed, the impact of the pro- and anti-inflammatory markers in any given situation is varied and depends upon many other factors, both qualitative and quantitative. The most important determinants for the inflammatory markers are their blood concentration, duration of expression, and the expressed level of their cell surface receptors. Therefore, any attempt to understand the role of a given cytokine by only assaying mRNA via real time PCR will not reveal the function of a cytokine in a given situation. Our measurements of circulating levels of inflammatory markers were essentially a “snapshot” of what would become a complex time course of events. Serum cytokines for diseased buffalo in the field may be present for extended periods, especially where the timescale for inflammation remains unknown. Our results indicated that TRP in buffalo was associated with higher serum levels of TNF-*α*, IL-1*β*, IL-6, and IL-10 and was reflected by increased concentrations of SAA, CRP, Hp, and Fb compared to the controls. It appears that the potent inhibitory action of IL-10 on IL-1, IL-6, TNF-*α*, and IF-*γ* supports the role of this cytokine in not only the regulation of T-cell responses, but also in acute inflammatory responses. IL-10 was also found to suppress the expression of IL-1, IL-6, and TNF-*α* protein for two macrophage cell lines *in vitro*, highlighting the role of IL-10 as a potent inhibitor of monokine synthesis [21]. The principal pathway leading to hepatic production of the APPs is the release of proinflammatory cytokines from macrophages at the site of inflammation [22]. IL-6, TNF-*α*, and IL-1*β* are key stimulators of hepatic APP production [13, 23, 24].

Serum SA levels have been analyzed in the literature for various bovine inflammatory and infectious diseases [16, 17], although SA concentration in buffalo with TRP has not previously been reported. In this study, diseased buffalo exhibited higher SA serum levels than the controls. The literatures have also reported that serum SA concentrations increase rapidly at the beginning of the inflammatory response or with injury. However, the underlying mechanism that causes such increase in serum SA has not been clearly outlined [17]. Some investigators have attributed the increase in SA levels to a rapid synthesis of sialoproteins and to an increase in globulins released from damaged tissue [16]. Moreover, the acute phase biochemistry may influence SA concentrations through the reactants' glycoprotein structure [25].

The quantification of blood APP levels can serve as both a diagnostic and prognostic tool where appropriate sampling timescales are realised [14]. Moreover, APP levels have been demonstrated in the literatures to be affected by a positive prognosis, but since the half-life of most APPs is only 24 to 48 hours, changes in APPs can become more sensitive indicators for healing and insult resolution than other clinical tests [8]. Several studies have previously denoted the significance of Fb, Hp, and CRP as useful biochemical parameters for evaluating the incidence and severity of inflammatory reactions in bovine species with inflammatory conditions [7, 9, 11, 26–30]. CRP was also found to be both a useful marker for evaluating the health status of a herd and a parameter to assess individual stress levels: CRP may be useful in early surveillance of intraherd TRP [29], as well as fulfilling an important function in defence against infection and control of the inflammatory response [31]. 

An adjunct tool for the diagnosis of TRP is USG, providing information with respect to the location and extent of the insult(s), and our findings were consistent with other reports [5, 6, 19]. However, contrary to other reports [32], our findings showed that buffalo with TRP had normal TLC, neutrophilia and lymphocytopenia (unlike the controls). The regenerative left shift in the leukogram is indicative of stress-related inflammation [20], and similar findings were reported by Hirvonen and Pyörälä [11].

## 5. Conclusion

The findings of this paper indicate that the examined immunologic variables were helpful in documenting the inflammatory response in buffalo with TRP. However, their usefulness for diagnosis only becomes apparent when considered in tandem with the clinical findings for any given animal, its anamnesis, and a subsequent USG evaluation.

## Figures and Tables

**Table 1:** Clinical findings in 22 buffalo with TRP compared to 10 controls.

Variables	Control (*n* = 10)	Diseased buffalo (*n* = 22)
Heart rate (bpm)	65.78 ± 2.63	78.00 ± 2.90^*^
Respiratory rate (breath/min)	11.00 ± 0.44	14.40 ± 0.50^**^
Rectal temperature (°C)	38.50 ± 0.18	39.26 ± 0.22^***^
Weakness and poor performance	Absent (*n* = 10)	Present (*n* = 3), absent (*n* = 19)
Abdominal distention	Absent (*n* = 10)	Present (*n* = 2), absent (*n* = 20)
Appetite	Normal (*n* = 10)	Normal (*n* = 0) Inappetence (*n* = 15) Anorexia (*n* = 7)
Ruminal motility	Normal (*n* = 10)	Normal (*n* = 0) Hypomotile (*n* = 7) Ruminal stasis (*n* = 15)
Recurrent tympany	Absent (*n* = 10)	Present (*n* = 21), absent (*n* = 1)
Lacrimation	Absent (*n* = 10)	Present (*n* = 17), absent (*n* = 5)
Lordosis/bruxism	Absent (*n* = 10)	Present (*n* = 6), absent (*n* = 16)
Changes in milk yield	Absent (*n* = 10)	Sharp declined (*n* = 18)∗

^*^ *P* < 0.0066, ^**^ *P* < 0.001, ^***^ *P* < 0.049. The remaining four patients were dry cows in late gestation.

**Table 2:** Means ± SEM of the leukogram in 22 buffalo with TRP compared to controls.

Variables	Control (*n* = 10)	Buffalo with TRP (*n* = 22)	*P* value
TLC (cell/*μ*L)	8,034 ± 310	7,730 ± 1.10	0.6461
Lymphocytes (cell/*μ*L)	4,186 ± 188	1,503 ± 0.530	0.0003
Neutrophils (cell/*μ*L)	3,424 ± 192	5,750 ± 1.40	0.0131
Band cells (cell/*μ*L)	0.3333 ± 0.2	277 ± 0.10	<0.0001
Monocytes (cell/*μ*L)	60.00 ± 6.427	178 ± 15	<0.0001
Eosinophils (cell/*μ*L)	54.20 ± 8.732	22 ± 0.18	0.0663

TLC: total leucocytic count.

**Table 3:** Means ± SEM of the selected inflammatory markers in buffalo with TRP compared to controls.

Variables	Control (*n* = 10)	Buffalo with TRP (*n* = 22)
TNF-*α* (pg/mL)	10.44 ± 0.56	22.30 ± 1.43∗
IL-1*β* (pg/mL)	18.00 ± 0.71	26.41 ± 1.69∗∗
IL-6 (pg/mL)	11.64 ± 0.68	31.12 ± 1.89∗
IL-10 (pg/mL)	10.15 ± 0.31	23.59 ± 1.93∗
IF-*γ* (pg/mL)	9.900 ± 0.18	10.28 ± 0.31^NS^
SAA (*μ*g/mL)	3.39 ± 0.41	100.5 ± 10.78∗
CRP (mg/L)	25.00 ± 1.73	354.3 ± 22.04∗
Hp (g/L)	0.114 ± 0.0138	2.24 ± 0.16∗
Fibrinogen (g/L)	4.07 ± 0.46	5.828 ± 0.23∗
Sialic acid (mmol/L)	0.162 ± 0.024	0.329 ± 0.025∗

TNF-*α*: tumour necrosis factor alpha; IL-1*β*: interleukin 1 beta; IL-6: interleukin 6; IF-*γ*: interferon gamma; SAA: serum amyloid A; CRP: C-reactive protein; Hp: haptoglobin. ^*^ *P* < 0.0001, ^**^ *P* < 0.0003, ^NS^nonsignificant, *P* = 0.3156.
